# Intraoperative vs. Postoperative Side-Effects-Thresholds During Pallidal and Thalamic DBS

**DOI:** 10.3389/fneur.2021.775784

**Published:** 2021-12-24

**Authors:** Victor J. Geraedts, Rogier A. P. van Ham, Jacobus J. van Hilten, Arne Mosch, Carel F. E. Hoffmann, Niels A. van der Gaag, Maria Fiorella Contarino

**Affiliations:** ^1^Department of Neurology, Leiden University Medical Center (LUMC), Leiden, Netherlands; ^2^Department of Clinical Epidemiology, Leiden University Medical Center (LUMC), Leiden, Netherlands; ^3^Department of Neurology, Haga Teaching Hospital, The Hague, Netherlands; ^4^Department of Neurosurgery, Haga Teaching Hospital, The Hague, Netherlands; ^5^Department of Neurosurgery, Leiden University Medical Center (LUMC), Leiden, Netherlands

**Keywords:** deep brain stimulation (DBS), GPi, Vim, monopolar contact review, intraoperative test stimulation

## Abstract

**Background:** It is currently unknown whether results from intraoperative test stimulation of two types of Deep Brain Stimulation (DBS), either during awake pallidal (GPi) or thalamic (Vim), are comparable to the results generated by chronic stimulation through the definitive lead.

**Objective:** To determine whether side-effects-thresholds from intraoperative test stimulation are indicative of postoperative stimulation findings.

**Methods:** Records of consecutive patients who received GPi or Vim were analyzed. Thresholds for the induction of either capsular or non-capsular side-effects were compared at matched depths and at group-level.

**Results:** Records of fifty-two patients were analyzed (20 GPis, 75 Vims). The induction of side-effects was not significantly different between intraoperative and postoperative assessments at matched depths, although a large variability was observed (capsular: GPi DBS: *p* = 0.79; Vim DBS: *p* = 0.68); non-capsular: GPi DBS: *p* = 0.20; and Vim DBS: *p* = 0.35). Linear mixed-effect models revealed no differences between intraoperative and postoperative assessments, although the Vim had significantly lower thresholds (capsular side-effects *p* = 0.01, non-capsular side-effects *p* < 0.01). Unpaired survival analyses demonstrated lower intraoperative than postoperative thresholds for capsular side-effects in patients under GPi DBS (*p* = 0.01), while higher intraoperative thresholds for non-capsular side-effects in patients under Vim DBS (*p* = 0.01).

**Conclusion:** There were no significant differences between intraoperative and postoperative assessments of GPi and Vim DBS, although thresholds cannot be directly extrapolated at an individual level due to high variability.

## Introduction

Deep Brain Stimulation (DBS) is an effective treatment to alleviate symptoms of various movement disorders, including Parkinson's disease (PD), dystonia, and Essential Tremor (ET), targeting either the subthalamic nucleus (STN), internal globus pallidus (GPi), or ventral intermediate nucleus of the thalamus (Vim) (1). Careful target localization is a prerequisite for successful surgery, which can be aided by microelectrode recording (MER) to identify target-specific electrical activity (2, 3). Test stimulation of the planned targets at several depths through the microelectrode macrostimulation tip is a common procedure in many centers to aid the placement of the definitive lead by evaluating the therapeutic effect of stimulation and the threshold for stimulation-induced side-effects (4). However, it is currently poorly understood whether results of this intraoperative test stimulation are comparable to the results post-operatively generated by chronic stimulation through the definitive lead.

In a previous study on patients with PD, we demonstrated that lower stimulation intensities were intraoperatively required than post-operatively to induce both relief of rigidity and induction of capsular side-effects, during STN DBS (5). These results were attributed to either differences in the design of the used electrodes, resulting in different Volume of Tissue Activated (VTA) and different current propagation within the VTA, or clinical condition (stun effect, medication withdrawal). Since data on targets, other than STN in awake surgeries, are lacking, it is still unclear whether our results are specific to the STN or are generalizable to other targets and indications.

In this study, we aimed to compare the thresholds for inducing side-effects between intraoperative test stimulation and postoperative chronic stimulation in Vim DBS and in GPi DBS. This knowledge could aid in clinical decision-making during the intraoperative choice of the best lead position and by potentially expediting the optimization of the postoperative DBS settings.

## Methods

A chart review was performed for all the consecutive patients receiving awake DBS for targets, other than STN (namely GPi and Vim), between September 2012 and February 2019 in the Haga/LUMC DBS center, and with available standard intraoperative and postoperative records. All patients were diagnosed with PD, dystonia, tremor, or a combination of these disorders. Given the retrospective nature of the study, a formal ethical evaluation was waived by the local medical ethics committee.

### Surgical Procedure

The surgical procedure was performed with standard DBS techniques; a detailed description of the surgical procedure is provided elsewhere (5). Lead-implantation was performed with patients while awake and without the use of sedatives. Pre-operatively performed stereotactic 3D MRI was used, in combination with StealthStation planning software (Medtronic, Minneapolis, Minnesota, USA), for localization of the target. Both posteroventral GPi and Vim targets were planned on the pre-operative T2, proton density scan (GPi), and T1 without contrast (Vim), starting from standard coordinates and adjusting the target based on individual anatomical landmarks. After intraoperative stimulation, the definitive lead for chronic stimulation was placed along the identified best trajectory based on the best clinical effect, and/or considering the threshold for debilitating (i.e., limiting) side-effects. The final position of the definitive lead was checked on intraoperative frame CT scans or postoperative (i.e., after 1 day) CT scans.

### Intraoperative Stimulation

Test stimulation was performed in awake patients with the microelectrodes macrostimulation tip (similar for all patients: electrodes FHC, Bowdoin Maine, USA), using a pulse width of 60 μs and a frequency of 130 Hz, with constant current at two to five depths, along each trajectory with at least 2 mm distance. Stimulation intensity was increased with steps of 0.5 mA, starting at 0.5–1.0 mA, until the first appearance of debilitating side-effects. If no side-effects occurred, test stimulation was discontinued after maximal 6 mA.

### Postoperative Stimulation

The position of the definitive lead was confirmed with intraoperative or postoperative CT-scans. The monopolar contact review was performed per clinical routine by the same neurologist who recorded the intraoperative results, ~10 days after surgery (range 9–10 days). Each of the four contact points of the definitive lead were tested in the same way as during intraoperative testing (constant current, pulse width 60 μs, and frequency 130 Hz); results of the intraoperative test stimulation were not used during the postoperative contact review. If no side-effects occurred, stimulation was usually discontinued after 6 mA, however higher stimulation intensities were occasionally tested at the neurologists' discretion. In case of directional leads, ring mode results were used for the purpose of this study.

### Outcome Measures

Side-effects were classified as either capsular or non-capsular. Capsular side-effects included muscle twitching and dysarthria; non-capsular side-effects included all other side-effects such as paresthesia, diplopia, phosphenes, nausea, and general discomfort. Paresthesia can arguably be considered as capsular as well, but were considered non-capsular given the stimulation in this particular region (6). Because it is often difficult to distinguish pure capsular dysarthria from dysarthria caused by stimulation of other structures, for the purpose of this study, we included all dysarthria under “capsular.” Gaze paresis was not consistently tested post-operatively and therefore was excluded from analyses. Side-effects were considered as “debilitating” when they were persistent and were severe enough to be unacceptable for chronic stimulation. Examples of debilitating side-effects were persistent dysarthria or muscle twitches. An example of non-debilitating side-effects was transient mild paraesthesia. Paraesthesia was considered transient if they disappeared (or considerably decreased in intensity) within a couple of minutes.

The therapeutic effect and the therapeutic window were not considered for this study, as therapeutic effect for dystonia is not evaluated during surgery. Intraoperatively tested depths, along with the finally chosen trajectory, were matched to corresponding postoperative contact points based on stereotactic target coordinates as previously described (5). For each stimulation point during the intraoperative stimulation, the distance from the pre-operatively defined target was recorded in 0.5 mm intervals on standardized forms. After test stimulation, the intraoperatively defined “best depth” was marked. The definitive lead used for chronic stimulation consists of four contact points measuring 1.5 mm and separated by 0.5 mm intervals. The tip of the definitive lead was mapped in the same way: this allowed matching of intraoperative stimulation depths to postoperative contact points with 0.5 mm accuracy. All available measurements were used during analysis.

### Statistical Analyses

Independent *t*-tests were used to compare thresholds between targets. Given the non-normal distribution, non-parametric Wilcoxon-signed rank tests were used to study matched pairs of intraoperative and postoperative assessments. Results were stratified per combination of target and type of side-effect. Linear mixed models, using a restricted maximum likelihood (REML), were used to model whether there are differences between intraoperative and postoperative assessments. The threshold for induction of debilitating side-effects was used as an outcome, whilst the timing of the testing (i.e., intraoperative vs. postoperative) was used as the “repeated” variable. Intra-individual variability was accounted for by modeling each individual as a random effect, whereas the influence of the anatomical target (i.e., Vim vs. GPi) was modeled as a fixed effect.

Comparisons at group-level were performed using Kaplan-Meier curves: an event was defined as the occurrence of a debilitating side-effect; censoring occurred if increasing the stimulation was discontinued without occurrence of side-effects. Separate curves were plotted for either intraoperative test stimulation or postoperative stimulation, as well as stratified per target. Log-rank (LR) tests were used to compare differences between the curves. Anonymized data may be shared upon request.

## Results

Fifty-six patients received DBS in targets other than STN. Three patients had missing records and were excluded; one patient received zona incerta DBS and was excluded as well. Fifty-two patients were ultimately included for analysis: 95 definitive leads were placed [86 bilateral and 9 unilateral; 77 model Medtronic 3389, 18 model Boston Scientific Vercise Cartesia (analyzed in ring-fashion)]. Patients' characteristics and targets are specified in Table 1.

**Table 1:** Patient characteristics.

	**Total**	**GPi**	**Vim**
N patients (leads)	52 (95)	21 (40)	31 (55)
Age at surgery^a^	59.8 (13.5)	51.6 (10.7)	65.4 (12.4)
Male sex^b^	61.5 (32)	42.9 (9)	74.2 (23)
Diagnosis^b^			
PD	28.8 (15)	14.3 (3)	38.7 (12)
Dystonia	32.7 (17)	81.0 (17)	0
Tremor	34.6 (18)	0	58.1 (18)
ET + Dystonia	1.9 (1)	0	3.2 (1)
PD + Dystonia	1.9 (1)	4.7 (1)	0
Post-operatively chosen electrode^b^, ^c^			
Most ventral	39.4 (39)	37.5 (15)	40.7 (24)
2nd-most ventral	43.4 (43)	45.0 (18)	42.4 (25)
2nd-most dorsal	16.2 (16)	17.5 (7)	15.3 (9)
Most dorsal	1.0 (1)	0	1.7 (1)

a *Mean (standard deviation)*.

b *% (n)*.

c *In 5 cases, the configuration was double monopolar, in 1 case bipolar and in all other cases monopolar*.

Post-operatively, both capsular (*p* < 0.001) and non-capsular (*p* < 0.001) side-effects occurred at lower thresholds in patients under Vim DBS than those under GPi DBS. Intraoperatively, non-capsular side-effects occurred at lower thresholds in patients under Vim DBS (*p* < 0.001); no difference was seen for capsular side-effects (*p* = 0.215).

### Induction of Side-Effects (Paired)

**Figure 1:**
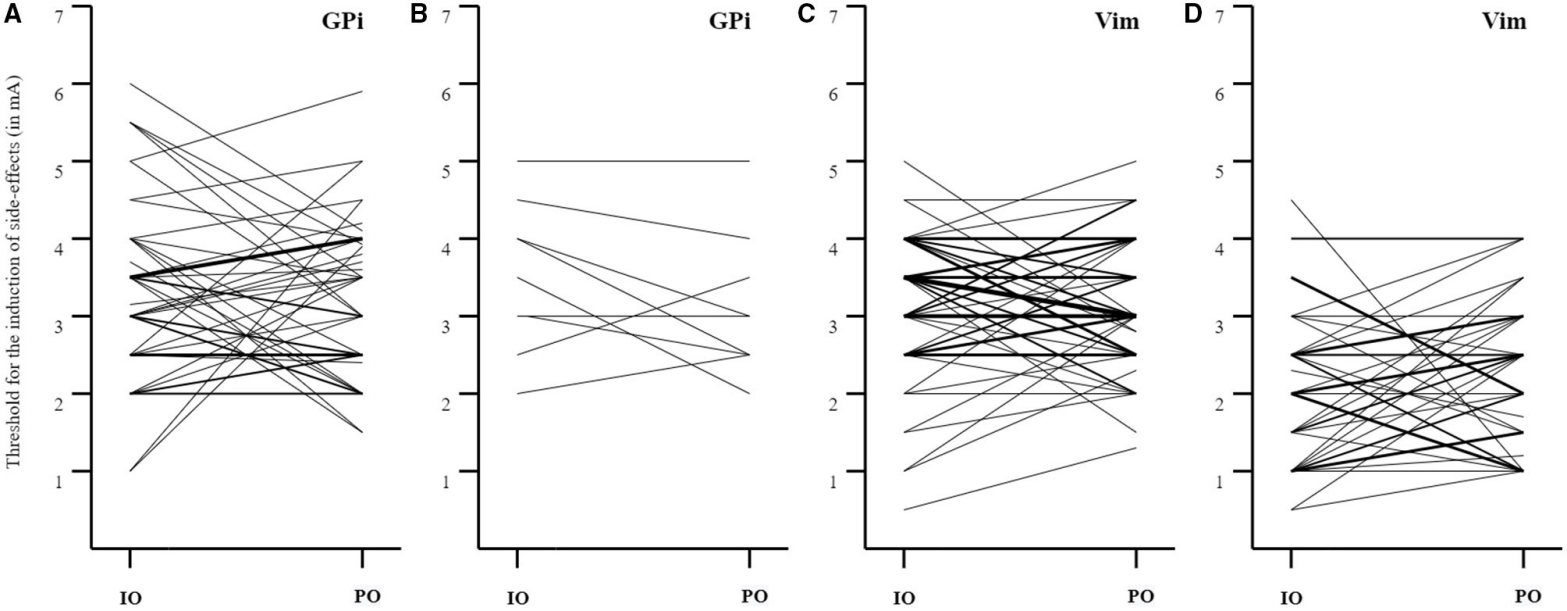
Paired analyses for the induction of capsular and non-capsular side-effects. **(A)** GPi DBS: induction of capsular side-effects (IO > PO: *n* = 20; IO < PO: *n* = 23; IO = PO: *n* = 5; mean ± SD threshold IO: 3.3 ± 1.1 mA, PO: 3.2 ± 1.0 mA, mean ± SD difference 1.0 ± 0.9 mA); **(B)** GPi DBS: induction of non-capsular side-effects (IO > PO: *n* = 5, IO < PO: *n* = 2, IO = PO: *n* = 2; mean ± SD threshold IO: 3.5 ± 1.0 mA, PO: 3.1 ± 0.9 mA, mean ± SD difference 0.6 ± 0.4 mA); **(C)** Vim DBS: induction of capsular side-effects (IO > PO: *n* = 23; IO < PO: *n* = 23; IO = PO: *n* = 14; mean ± SD threshold IO: 3.2 ± 0.9 mA, PO: 3.1 ± 0.8 mA, mean ± SD difference 0.8 ± 0.6 mA); **(D)** Vim DBS: induction of non-capsular side-effects (IO > PO: *n* = 14; IO < PO: *n* = 26; IO = PO: *n* = 7; mean ± SD threshold IO: 2.1 ± 1.0 mA, PO: 2.3 ± 0.9 mA, mean ± SD difference 0.9 ± 0.6 mA). The thickness of the lines reflects the number of paired observations. IO, intraoperative; PO, postoperative.

The threshold for induction of capsular side-effects was not significantly different between paired intraoperative and postoperative assessments for patients under GPi DBS and Vim DBS (GPi DBS: 48 pairs, *Z* = −0.27, *p* = 0.79; Vim DBS: 60 pairs, *Z* = 0.42, *p* = 0.68). Similarly, the induction of non-capsular side-effects was not significantly different either for both targets (GPi DBS: 9 pairs, *Z* = −1.28, *p* = 0.20; Vim DBS: 47 pairs, *Z* = −0.94, *p* = 0.35). Although the paired stimulation intensities did not significantly differ between the assessments, Figure 1 demonstrates the variability between assessments.

For both capsular and non-capsular side-effects, linear mixed models showed that there was no significant difference between the intraoperative and postoperative thresholds, while accounting for intra-individual variability and target (capsular side-effects: β = −0.04*, p* = 0.80; non-capsular side-effects: β = −0.00, *p* = 0.98). The target significantly contributed to the threshold level-prediction (capsular side-effects: β = 0.44, *p* = 0.01; non-capsular side-effects: β = 1.08, *p* < 0.01), with thalamic stimulation having lower thresholds.

### Induction of Side-Effects (Overall)

**Figure 2:**
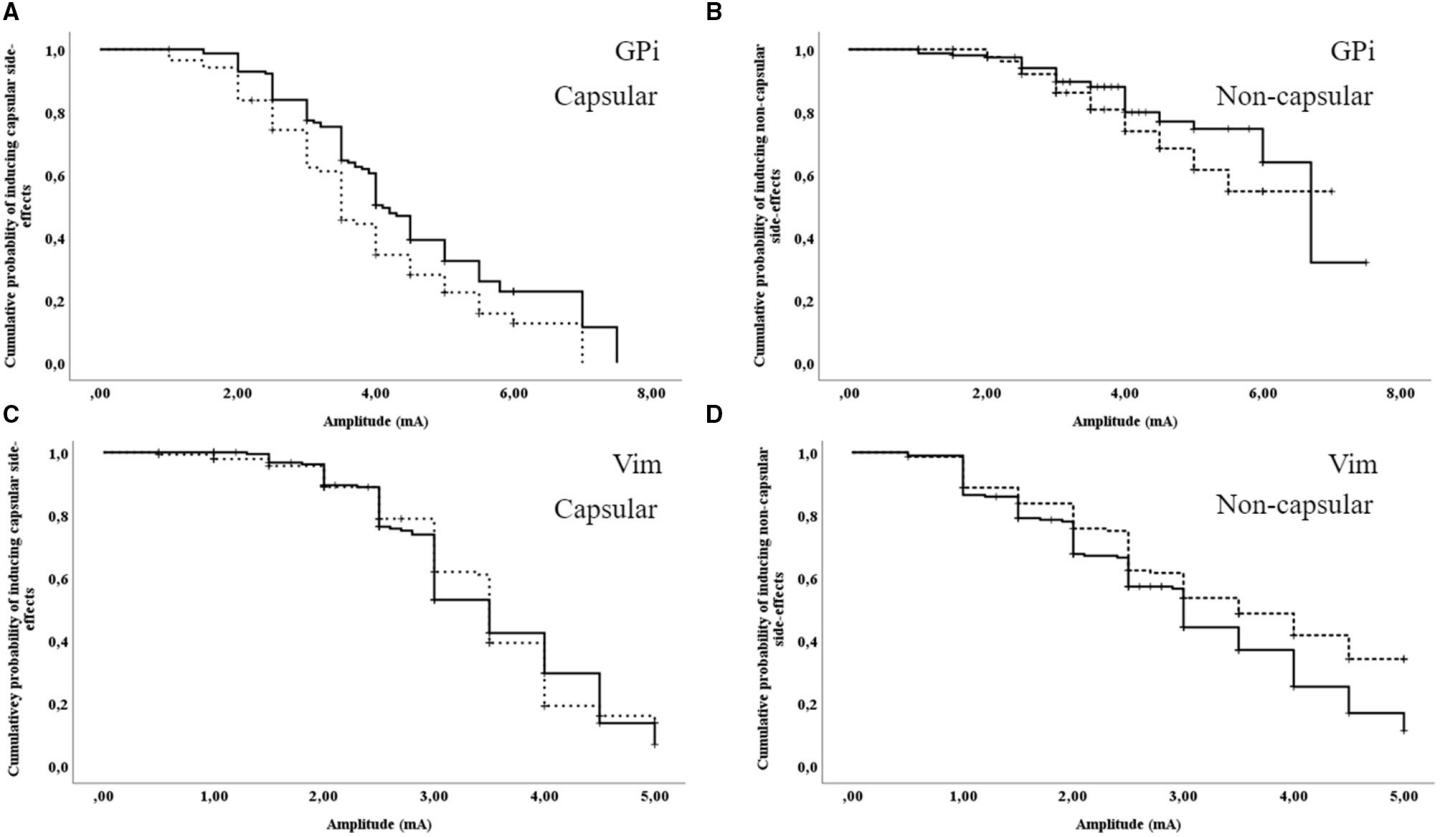
Unpaired survival analyses for the induction of capsular and non-capsular side-effects. **(A)** GPi DBS: induction of capsular side-effects; **(B)** GPi DBS: induction of non-capsular side-effects; **(C)** Vim DBS: induction of capsular side-effects; **(D)** Vim DBS: induction of non-capsular side-effects. Dashed line: intraoperative assessment; continuous line: postoperative assessment. Vertical ticks indicate censoring.

With patients in GPi DBS [69 intraoperative events (17 censored data points) vs. 99 postoperative events (57 censored data points)], capsular side-effects were induced at a significant lower threshold during intraoperative stimulation than during postoperative stimulation in unpaired survival analyses (intraoperative mean (95%CI) 3.90 (3.54–4.27) mA vs. postoperative mean (95%CI) 4.58 (4.25–4.91) mA, χ^2^ = 6.17, *p* = 0.01, see Figure 2A). For non-capsular side-effects [21 intraoperative events (65 censored data points) vs. 30 postoperative events (126 censored data points)], unpaired survival analyses showed no significant difference [intraoperative mean (95%CI) 5.62 (5.14–6.10) mA vs. postoperative mean (95%CI) 6.06 (5.56–6.57) mA, χ^2^ = 1.69, *p* = 0.19, see Figure 2B].

With patients in Vim DBS [98 intraoperative events (45 censored data points) vs. 119 postoperative events (72 censored data points)], capsular side-effects did not significantly differ between intraoperative and postoperative assessments in unpaired survival analyses [intraoperative mean (95%CI) 3.48 (3.31–3.66) mA vs. postoperative mean (95%CI) 3.49 (3.34–3.65) mA, χ^2^ = 0.03, *p* = 0.87, see Figure 2C]. For non-capsular side-effects [75 intraoperative events (68 censored data points) vs. 124 postoperative events (67 censored data points)], unpaired survival analyses demonstrated a higher intraoperative threshold than post-operatively [intraoperative mean (95%CI) 3.43 (3.19–3.68) mA vs. postoperative mean (95%CI) 3.05 (2.85–3.26) mA, χ^2^ = 6.00, *p* = 0.01, see Figure 2D].

## Discussion

In this study, we compared thresholds for inducing side-effects during intraoperative test stimulation to thresholds observed during the postoperative monopolar contact review with patients in both GPi and Vim DBS.

Our results demonstrate no significant difference between intraoperative and postoperative assessments for the induction of both capsular and non-capsular side-effects, for both patients of GPi and Vim DBS, assessed with either Wilcoxon-signed rank tests for paired observations or linear mixed effect models accounting for intra-individual variability. However, although the results may not differ at group-level, individual variability in the range of 0.5–3.5 mA in either direction is evident, rendering the results from the intraoperative assessment inaccurate for the prediction of postoperative thresholds at the individual level. We therefore recommend maintaining the postoperative monopolar contact review in full, to record reliable thresholds for side-effects as reference point for chronic stimulation. Although intraoperative testing along the best trajectory appears inaccurate in predicting the threshold of postoperative effects in patients under Vim and GPi DBs, this is still relevant for target-positioning when comparing different trajectories. In this view, our results therefore do not disqualify the utility of intraoperative testing.

Overall, in unpaired analyses, lower thresholds were observed in patients under GPi DBS during intraoperative test stimulation for the induction of both capsular and non-capsular side-effects than during postoperative stimulation, although the latter did not reach statistical significance. In contrast, no differences were seen in patients under Vim DBS in terms of capsular side-effects, whereas higher stimulation intensities were observed to induce non-capsular side-effects than during postoperative stimulation. This analysis was performed to compare our results with previous studies that adopted this method (5, 7), which accounts for censored data and, as it does not correct for matched depths, keeps the sample size largely intact. However, in this analysis, the significance of the results may be influenced by the number of observations and by other factors implicit to the nature of the data collection. For example, the most dorsal points along the best trajectory in the GPi (i.e., more distant from the internal capsule and deeper structures, thus, having a higher threshold for side-effects) are often not tested during surgery when the deepest points have sufficiently high thresholds. On the opposite, all points are tested during the postoperative contact review. Also, the “best trajectory” chosen for chronic stimulation in all targets is usually the trajectory with the highest threshold for side-effects, thus the postoperative measurements are again more likely to have higher thresholds for inducing side-effects than the other trajectories explored during the entire intraoperative assessment.

Combined with results from our previous study on patients under STN DBS, which demonstrated lower intensities during intraoperative stimulation for capsular side-effects (5), it appears that there different targets behave differentially when comparing intraoperative and postoperative thresholds (8, 9). These results can hardly be explained by the difference in electrode (i.e., microelectrode vs. definitive lead), considering that this condition was the same across targets. Less likely to contribute to the differences and /or variability in required stimulus intensity between assessments across targets is the size of the VTA (10), the degree of encapsulation of the chronic electrode (11), lead displacement or brain shift (12), which should also not differ greatly between targets. The potential role of these mechanisms in determining the individual variability of intraoperative and postoperative thresholds could not be explored further in this study. Further consideration should be given to the time-point of postoperative assessments, as the initial monopolar contact review may not be entirely representative of the long-term follow-up DBS settings (5, 13). In our experience, a stun effect is negligible in the majority of patients 10 days after surgery, nevertheless we cannot exclude that a potential partial stun effect may have been present in some cases. Although this would not be enough to explain the observed difference between the targets, and, although, our study focused on the threshold for side-effects, which is notably less affected by the stun effect, a variable degree of stun effect could have accounted for part of the individual variability.

A previous study on dystonic patients under GPi DBS (*n* = 6) has demonstrated a trend toward higher intraoperative stimulation intensities required for induction of side-effects. However, this study assessed intraoperative stimulation under general anesthesia, rendering the setting to study the induction of side-effects altogether different (13).

None of the used statistical methods show a full unbiased overview. Paired assessments matched for depth consider anatomical variations at an individual level, but do not consider censored data thereby leading to a substantial loss of data. Linear mixed effect models consider the intra-individual variability but are not matched for depth. In contrast, unpaired survival analyses consider censored data but do not account for matching of depth, thereby keeping the sample size intact, but disregarding some of the anatomical variations of the target and surrounding structures. Results are therefore best interpreted jointly.

Strengths of this study include the large sample size of studied targets, the standardized procedures, and the systematic recording of both capsular and non-capsular side-effects. The consistency of our approach (i.e., the same neurologists performed both the intra- and postoperative assessments) constitutes a clear strength, although some bias about how side-effects were detected and recorded may have been introduced as well. Limitations include the retrospective design and inherent missing data. Despite the retrospective design, our method does reflect a systematic clinical approach which was consistent across involved neurologists. The number of missing data may have led to some bias, as both intraoperative and postoperative assessments at several depths and intensities were made at the physician's discretion, while data was, therefore, to be considered missing-not-at-random (MNAR). Inter-rater variability was not addressed as most of our patients was assessed by the same neurologist and the remaining data was insufficient to address this topic. Imputation of missing data was considered unfeasible given the large amount of missing data relative to observed data, and the missingness-structure being MNAR.

Future research should apply prospective designs to avoid missing data. Exploration of the reasons behind the individual variability may provide further insight into the mechanisms behind the discrepancies between the various analyses. Studies focusing on prediction models, possibly applying automatic programming algorithms, should be performed to investigate whether prediction of optimal DBS settings for chronic stimulation can be performed based on intraoperative assessments.

## Data Availability Statement

The raw data supporting the conclusions of this article will be made available by the authors, upon reasonable request

## Ethics Statement

Ethical review and approval was not required for the study on human participants in accordance with the local legislation and institutional requirements. Written informed consent for participation was not required for this study in accordance with the national legislation and the institutional requirements.

## Author Contributions

VG and MFC designed and conducted the study. AM, CH, NG, and MFC performed patient recruitment and data collection. VG and RH performed data analysis and statistical analysis. VG and RH prepared the manuscript draft with important intellectual input from AM, CH, JH, NG, and MFC. VG, RH, and MFC had complete access to the study data. All authors critically reviewed the final manuscript and approved the final manuscript.

## Conflict of Interest

Unrelated to this study, JH reports grants from The Netherlands Organisation for Health Research and Development, The Netherlands Organisation for Scientific Research, Hoffmann-La Roche, AbbVie, Lundbeck, Hersenstichting, Stichting Parkinson Fonds, Alkemade-Keuls Foundation, and Centre of Human Drug Research. AM reports travel support from Boston Scientific. CH reports travel support from Boston Scientific. NG reports travel support from Boston Scientific. MC reports support for advisory board from Medtronic (fees to institution), consultancy fees: Medtronic (fees to institution), CHDR (fees to institution), research support: Medtronic (to institution), AbbVie (to institution), research support in kind from Global Kinetics Corporation, travel support: Boston Scientific. The remaining authors declare that the research was conducted in the absence of any commercial or financial relationships that could be construed as a potential conflict of interest.

## Publisher's Note

All claims expressed in this article are solely those of the authors and do not necessarily represent those of their affiliated organizations, or those of the publisher, the editors and the reviewers. Any product that may be evaluated in this article, or claim that may be made by its manufacturer, is not guaranteed or endorsed by the publisher.
